# Everyday Executive Functioning in Paediatric Patients Awaiting Solid Organ or Haematopoietic Stem Cell Transplantation

**DOI:** 10.1192/j.eurpsy.2025.1687

**Published:** 2025-08-26

**Authors:** J. Garrido-Bolton, M. F. Bravo-Ortiz, R. de la Vega, A. Pérez-Martínez, E. Fernández-Jiménez

**Affiliations:** 1 Department of Psychiatry, Clinical Psychology and Mental Health, La Paz University Hospital; 2 Hospital La Paz Institute for Health Research (IdiPAZ); 3 Department of Psychiatry, Universidad Autónoma de Madrid (UAM); 4 Instituto de Salud Carlos III, Centro de Investigación Biomédica en Red de Salud Mental (CIBERSAM), Madrid; 5 University of Málaga, Department of Personality, Evaluation and Psychological Treatment; 6 Instituto Biomédico de Málaga – IBIMA Plataforma Bionand, Málaga; 7 Department of Pediatric Hemato-Oncology, La Paz University Hospital; 8 Spanish National Cancer Research Center (CNIO), CIBERER-ISCIII. IdiPAZ-CNIO Pediatric Onco-Hematology Clinical Research Unit; 9 Department of Pediatrics, Universidad Autónoma de Madrid (UAM), Madrid; 10 Faculty of Social Sciences and Communication, Universidad Europea de Madrid, Villaviciosa de Odón, Spain

## Abstract

**Introduction:**

Paediatric patients with severe diseases awaiting solid organ or haematopoietic stem cell transplantation are known to experience cognitive impairments, particularly in executive functioning. Neuropsychological pre-transplant evaluation serves as a baseline for identifying executive functioning deficits that may affect the medical and psychosocial aspects of care. This is clinically relevant because patients with poorer executive functioning may show decreased adherence to medical treatments, face greater challenges in coping with their illness, and be more likely to require educational adaptations, including curricular or methodological adjustments based on pedagogical criteria.

**Objectives:**

This study aimed to assess everyday executive functioning in paediatric patients awaiting transplantation using an ecologically valid measure.

**Methods:**

A total of 49 patients (59.2% male) aged between 6 and 18 years (*M* = 11.4, *S.D.* = 3.5) were recruited from La Paz University Hospital (Madrid, Spain). Patients were awaiting for various types of organ transplants (kidney: 26, heart: 4, lung: 4, hepato-renal: 3, or liver: 2 patients) or haematopoietic stem cells (10 patients). Patients were assessed using the Spanish parent-reported version of the *Behavior Rating Inventory of Executive Function, Second Edition* (BRIEF-2) with age- and sex-adjusted norms from the Spanish general population. The three BRIEF-2 composite scores were analysed (the Cognitive, Emotional and Behavioral Regulation Indexes), and clinically relevant scores were set at *T* ≥ 65 points.

**Results:**

Clinically significant levels of executive deficits were observed in 33.3% of patients regarding cognitive regulation problems, in 32.7% regarding emotional regulation difficulties and in 26.5% regarding behavioural regulation problems.

**Conclusions:**

Between 1 in 4 and 1 in 3 patients have shown some type of executive regulation difficulties. The early identification of executive functioning deficits in paediatric transplant candidates is crucial. Incorporating standardised ecologically valid measures, such as the BRIEF-2, into routine assessments can help detect neuropsychological impairments at an early stage, allowing timely therapeutic or preventive interventions (e.g., psychological prehabilitation). This approach can improve medical outcomes and quality of life and guide educational adaptations to support academic performance.

**Disclosure of Interest:**

None Declared

